# Complete hemispherotomy leads to lateralized functional organization and lower level of consciousness in the isolated hemisphere

**DOI:** 10.1002/epi4.12433

**Published:** 2020-09-24

**Authors:** Thomas Blauwblomme, Athena Demertzi, Jean‐Marc Tacchela, Ludovic Fillon, Marie Bourgeois, Emma Losito, Monika Eisermann, Daniele Marinazzo, Federico Raimondo, Sarael Alcauter, Frederik Van De Steen, Nigel Colenbier, Steven Laureys, Volodia Dangouloff‐Ros, Lionel Naccache, Nathalie Boddaert, Rima Nabbout

**Affiliations:** ^1^ Assistance Publique Hôpitaux de Paris Hôpital Necker‐Enfants Malades Paris France; ^2^ Université de Paris Paris France; ^3^ INSERM U1163 Institut Imagine Paris France; ^4^ GIGA‐Consciousness, Physiology of Cognition Research Lab GIGA Institute University of Liège Liège Belgium; ^5^ INSERM U1127 Paris France; ^6^ Institut du Cerveau et de la Moelle Epinière Hôpital Pitié‐Salpêtrière Paris France; ^7^ Department of Data Analysis Faculty of Psychological and Educational Sciences University of Ghent Ghent Belgium; ^8^ GIGA‐Consciousness, Coma Science Group GIGA Institute University of Liège Liège Belgium; ^9^ Instituto de Neurobiología Universidad Nacional Autónoma de México Querétaro México

**Keywords:** arterial spin labelling, consciousness, functional MRI, hemispherotomy, networks

## Abstract

**Objective:**

To quantify whole‐brain functional organization after complete hemispherotomy, characterizing unexplored plasticity pathways and the conscious level of the dissected hemispheres.

**Methods:**

Evaluation with multimodal magnetic resonance imaging in two pediatric patients undergoing right hemispherotomy including complete callosotomy with a perithalamic section. Regional cerebral blood flow and fMRI network connectivity assessed the functional integrity of both hemispheres after surgery. The level of consciousness was tested by means of a support vector machine classifier which compared the intrinsic organization of the dissected hemispheres with those of patients suffering from disorders of consciousness.

**Results:**

After hemispherotomy, both patients showed typical daily functionality. We found no interhemispheric transfer of functional connectivity in either patient as predicted by the operation. The healthy left hemispheres displayed focal blood hyperperfusion in motor and limbic areas, with preserved network‐level organization. Unexpectedly, the disconnected right hemispheres showed sustained network organization despite low regional cerebral blood flow. Subcortically, functional connectivity was increased in the left thalamo‐cortical loop and between the cerebelli. One patient further showed unusual ipsilateral right cerebello‐cortical connectivity, which was explained by the mediation of the vascular system. The healthy left hemisphere had higher probability to be classified as in a minimally conscious state compared to the isolated right hemisphere.

**Significance:**

Complete hemispherotomy leads to a lateralized whole‐brain organization, with the remaining hemisphere claiming most of the brain's energetic reserves supported by subcortical structures. Our results further underline the contribution of nonneuronal vascular signals on contralateral connectivity, shedding light on the nature of network organization in the isolated tissue. The disconnected hemisphere is characterized by a level of consciousness which is necessary but insufficient for conscious processing, paving the way for more specific inquiries about its role in awareness in the absence of behavioral output.

**Key Point**

- Complete hemispherotomies are rare operations permitting the evaluation of functional processing in both hemispheres as compared to other surgical procedures
- After hemispherotomy, no interhemispheric transfer was identified in two pediatric patients evaluated with multimodal MRI
- Cortical connectivity was lateralized, with preserved network organization in the defected right hemisphere despite hypoperfusion
- Thalamo‐cortical and cerebello‐cortical connectivity was preserved in the healthy hemispheres; intercerebellar connectivity was unaffected
- The defected right hemisphere resembled less the conscious capacities of patients in minimally conscious state

## 1 INTRODUCTION

Ηemispherotomy is the surgical disconnection of the two cerebral hemispheres as a treatment for intractable epilepsy. In pediatric populations, hemispherotomies lead to a 70% seizure freedom rate and a good functional outcome.^1^, ^2^ Despite living with only one hemisphere, operated children regain at least a partial sensory motor function, do not worsen their cognitive skills, and may recover from language deficits regardless of the operated side.^3^, ^4^ In terms of brain plasticity, diverse medical imaging and electrophysiological techniques (transcranial magnetic stimulation,^5^ somatosensory evoked potentials, positron emission tomography,^6^ functional magnetic resonance imaging (fMRI),^7^, ^8^ tensor diffusion‐weighted imaging,^9^ and combination of these techniques^10^) point to minimal motor plasticity changes in the remaining hemisphere, structural deteriorations in the affected hemisphere, and the ability to transfer motor and sensory functions from the affected hemisphere to the remaining one.^7^, ^11^ Collectively, these studies have advanced our understanding about specific cerebral mechanisms after hemispherotomies. A whole‐brain quantification of cerebral functional organization, though, is expected to inform about unexplored plasticity pathways, especially after complete hemispherotomies which target the dissection of interhemispheric association bundles, projection as well as thalamo‐cortical fibers.^12^

Additionally, hemispherotomies continue to raise scientific questions. The scientific concerns refer to whether functional organization in causally isolated brain tissue can support conscious states that are neither shaped by sensory input nor able to be expressed by motor output (ie islands of awareness).^13^ So far, this issue has been primarily addressed with studies with callosotomies leading to well‐known split‐brain cases.^14^ Collectively, these studies show that integrated information between the two hemispheres breaks down such that one hemisphere is not conscious of what the other one is perceiving and thinking^14^, ^15^—patients, though, continue to experience themselves in an integrated manner.^16^, ^17^ Split‐brain cases also show that network functional connectivity remains bilateral, with interhemispheric correlations either falling within typical range,^18^, ^19^ or showing significantly reduced yet preserved cortico‐cortical and thalamo‐thalamic correlations.^20^ Similar patterns of bilaterally symmetric networks have been also reported for ﻿patients with complete agenesis of the corpus callosum.^19^ Although callosotomies offer ample knowledge about the integrative role of corpus callosum in cognition, they nevertheless do not allow for a comprehensive characterization of the relationship between cerebral structure and function as they do not ensure complete rupture of cortical information exchange between the two hemispheres.^21^, ^22^

Driven by the clinical and scientific imperative, we here aim at quantifying whole‐brain functional organization after hemispherotomy with an additional examination of the consciousness levels of the disconnected hemispheres. Consciousness level refers to the organism's overall conscious condition,^23^ ranging from alert wakefulness to postcomatose conditions, such as the unresponsive wakefulness syndrome/vegetative state^24^ (UWS/VS), and states that are associated with light‐to‐moderate degrees of sedation, dreaming, and absence seizures.^13^ Using postoperative multimodal functional neuroimaging in two pediatric patients suffering from intractable epilepsy, we hypothesized that (a) functional organization will appear lateralized, consistent with the surgical procedure, (b) subcortical structures will appear as critical relays accounting for neurological stability, and (c) the disconnected hemisphere would be characterized by a low level of consciousness. For those purposes, we respectively opted for (a) whole‐brain fMRI contralateral connectivity assessment as well as quantification of blood perfusion, (b) hypothesis‐driven connectivity analysis of the thalami and cerebelli, and (c) a classification scheme of each patient's hemispheres with those of patients with disorders of consciousness.

## 2 MATERIAL AND METHODS

### 2.1 Subjects and acquisition protocol

**FIGURE 1:**
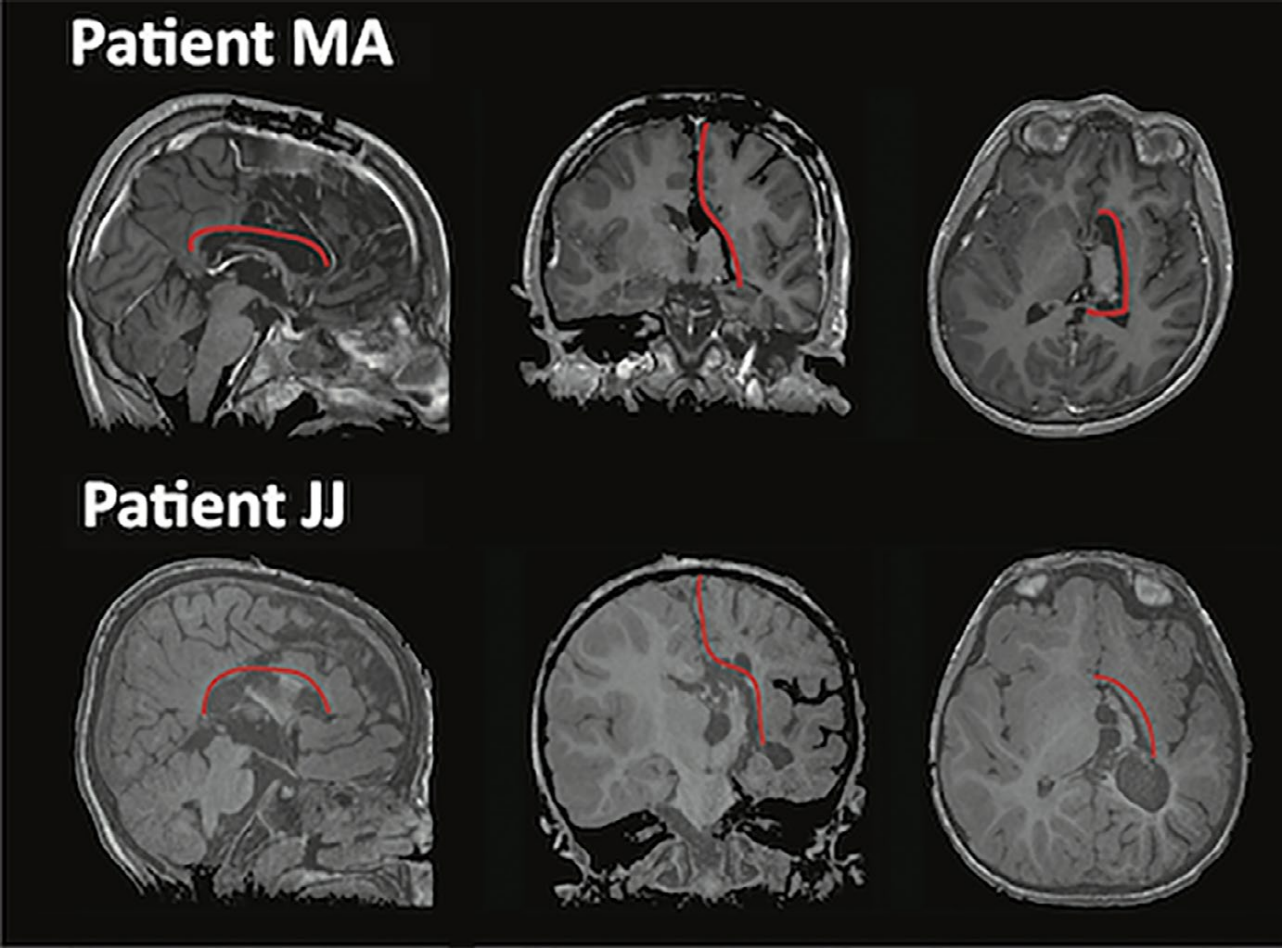
Surgical disconnection of the pathological right hemisphere in a case of Rasmussen's encephalitis (patient MA) and hemimegalencephaly (patient JJ). The red line shows the surgical perithalamic disconnection after a midline approach: after an interhemispheric section, complete callosotomy was performed allowing access to the lateral ventricles. Perithalamic section of the white matter between the frontal and temporal horn disrupted the internal capsule, fimbria, anterior commissure, but left the major intra‐hemispheric bundles (superior and inferior longitudinal fasciculi, uncinated fasciculus, cingulum, external capsule) untouched

Ten hemispherotomies were performed between 2013 and 2014. Eight patients accepted to be enrolled in the “CREIM imaging protocol” approved by the Necker Hospital local ethics committee. Six of these patients were excluded due to excessive motion during the MRI acquisition obstructing the scanning session; ongoing seizure activity in the disconnected hemisphere; or behavioral problems precluding full protocol without sedation. The two included patients were operated by a midline vertical hemispherotomy^12^ (Table 1). A senior radiologist (NB) confirmed the anatomical perithalamic disconnection on T1 MRIs, including disruption of corpus callosum, anterior commissure, fornix, and internal capsule (Figure 1). The MRI scanning concerned functional MRI (fMRI) and arterial spin labeling (ASL) acquisitions. The MRI protocol was performed without sedation 39 and 31 months after surgery for MA and JJ, respectively.

**TABLE 1:** Patient demographic characteristics

	MA	JJ
Gender	male	male
Etiology	Rasmussen encephalitis	Hemimegalencephaly
Age at Sz onset	13.6y	10d
Sz semiology	Epilepsia Partialis Continua	Spasms
Age at surgery (yrs)	14.3	2
Age at MRI (yrs)	17.6	4.6
Preoperative EEG	PC central spikes	PC R temporooccipital spikes
Laterality	Right handed	NA
Seizure outcome	Engel score Ia, 5 y	Engel score Ia, 6 y
Motor outcome 1	Hemi‐paresis, hemi‐anopia, hypertonia	Hemi‐paresis, hemi‐anopia, hypertonia
Motor outcome 2	Walks, runs, eats, draws	Walks, runs, eats, draws
Functionality	Typical high school, 3 languages	Typical nursery school, bilingual

Abbreviations: PC, pseudocontinuous; Sz, seizure.

For the fMRI session, data were acquired on a GE Discovery MR750 3T system and included 300 functional MRI T2*‐weighted images acquired with a gradient‐echo echo‐planar imaging (EPI) sequence using transverse slice orientation and covering the whole brain (39 slices, slice thickness = 3 mm, repetition time = 2000 ms, echo time = 34 ms, voxel size = 3.125 × 3.125 mm, flip angle = 90°). A structural T1 magnetization prepared rapid gradient‐echo sequence (120 slices, repetition time = 2300 ms, echo time = 2.47 ms, voxel size = 1.0 × 1.0 × 1.2 mm, flip angle = 9°). Healthy controls were included as a reference in the second‐level statistical model. These healthy subjects were age‐matched and obtained from the National Database for Autism Research (NDAR) (http://ndar.nih.gov) scanned on 3T scanners (Siemens Magnetom TrioTim or General Electric SignaHDxt). The dataset for patient MA included n = 11 controls (1 female, mean age = 16.8 years ± 0.5SD, min = 16, max = 18). The dataset for patient JJ included n = 9 controls (4 females, mean age = 3.2 years ± 0.3SD, min = 3, max = 4).

For the ASL data, the 3D ASL sequences were acquired on a GE Signa HDxt 1.5T system (General Electric Medical System, Milwaukee, USA) using a twelve‐channel head‐neck‐spine coil including morphological sequences (3D T1‐weighted images, axial T2 FLAIR, diffusion) noncontrast perfusion imaging with 3D pseudocontinuous ASL MRI (pcASL). The acquisition included 80 axial partitions (field of view 240 × 240 × 4 mm^3^; acquisition matrix 8 spiral arms in each 3D partition, 512 points per arm; TE 10.5 ms; TR 4428 ms; postlabeling delay 1025 ms; flip angle 155°; acquisition time 4 min 17 s). For the ASL analysis, 30 healthy pediatric controls were used as previously reported.^25^

### 2.2 fMRI preprocessing and connectivity analysis

Preprocessing was performed with SPM12 (slice‐time correction, realignment, segmentation of structural data, normalization of functional and structural data into standard stereotactic MNI space, and spatial smoothing using a Gaussian kernel of 6 mm full‐width at half‐maximum). The three initial functional volumes were discarded to avoid T1 saturation effects. Motion artifact correction was performed with the artifact detection toolbox (ART toolbox, www.nitrc.org/projects/artifact_detect): outliers were images with head displacement >2 mm from the previous frame, or rotational displacement >0.02 radians from the previous frame, or global mean intensity >3SD from the mean image intensity for the entire session. Outliers were subsequently included as nuisance regressors within the first‐level general linear model. Denoising followed the anatomical component‐based noise correction method^26^ as implemented in CONN v.17f^27^: each subject's white matter (WM) and cerebrospinal fluid masks (CSF) were used to derive the time series from the unsmoothed functional volumes and performed principal component analysis. The first five principal components were regressed out. The default mask as provided by the toolbox was used for patient MA. Due to the developing brain morphology of patient JJ, a template mask of a two‐year old was used: the template was constructed with longitudinal and cross‐sectional group‐wise registrations of a set of images acquired from 95 typical infants. For the sake of the current analysis, the two‐year‐old group‐wise anatomical (intensity) model with skull was used.^28^ To minimize partial voluming with gray matter (GM), the WM and CSF masks were eroded by one voxel which resulted in smaller masks than the original segmentations. Residual head motion parameters were further regressed out. A temporal band‐pass filter [0.008‐0.09 Hz] was applied on the time series to restrict the analysis to low‐frequency fluctuations.

#### 2.2.1 Interhemispheric correlations

Interhemispheric correlations were estimated using Pearson's *r* between each subject's right and left GM averaged time series. The segmented smoothed GM images were separated between the left and the right hemisphere using the fsl_roi function. These half‐hemisphere GM images were used as masks to extract averaged time series from each subject's denoised data and the Pearson's *r* coefficient was calculated.

#### 2.2.2 Functional connectivity

Functional connectivity analyses were performed in standard space in order to obtain whole‐brain correlation maps at the second level. The analysis adopted a seed‐based correlation approach with manually designed ROIs following each patient's anatomical constraints after normalization (Tables S1 and S2). The ROIs were 5‐mm‐radius spheres referring to pertinent intrinsic connectivity networks, such as the default mode, frontoparietal, salience, motor, auditory, and visual.^29^, ^30^, ^31^, ^32^ Due to the young age of patient JJ, the ROI for left thalamus with connections to DMN followed the coordinates from our previous work.^33^ The ROIs‐T1 registration accuracy is visualized by means of the quality assurance plots as implemented in Conn and is summarized in Figure S5 (MA) and Figure S6 (JJ).

For each patient, a separate Conn project was created including the patient and his corresponding control subjects. To test for contralateral network connectivity, the used seeds were located either in the left or in the right hemisphere (Table S1). The averaged time series within each seed ROI was used to estimate whole‐brain correlation r maps which were converted to normally distributed Fisher's z‐transformed maps to allow for group‐level comparisons. One‐sample *t* tests estimated network‐level connectivity for patients MA and JJ separately using their corresponding control subjects as a reference group [modeling 1(patient) 0 (controls)]. To allow for identification of potential contralateral connectivity, we favored the risk of type 1 error^34^ and the results were considered significant at a liberal height threshold *P* < .05, with cluster‐level parametric correction for multiple comparisons at family‐wise error rate (FWE) *P* < .05.

#### 2.2.3 Effect of vascularization

Considering that the vascular system is the major physiological system shared by the two hemispheres, we opted to isolate the effect of vascularization on the BOLD neuronal signal. Indeed, systemic low‐frequency oscillations (sLFO ~ 0.1 Hz) are usually present in vascularized tissue with cardiac, respiratory, and peripheral origin and are included in the BOLD fMRI variance.^35^ The sLFO, especially in large veins such as the superior sagittal sinus (SSS), were shown to highly correlate with the fMRI global signal.^36^, ^37^ Using the 3Dslicer (v4.8.1 r26813)^38^ on the patient's T1/T2 or FLAIR raw data, we identified the SSS manually. The identified segments were then coregistered and normalized in MNI space to match the dimensions of the normalized functional images. The extracted time series from the SSS were then used as a noise ROI to regress out the SSS effect. The statistical associations between the SSS signal and the rest of the brain are summarized in Figure S1 and Figure S2.

#### 2.2.4 Classification of consciousness level

The assessment of consciousness level was tested by a modified version of a previously developed classifier targeting to separate patients in minimally conscious state (MCS, showing complex behavioral responses to external stimulation, such command following and pursuit of moving objects) from patients in UWS (showing reflexive behaviors).^29^ The classification pattern referred to a binary mask containing bilateral superior temporal/precentral gyri and occipital areas. Here, the pattern was normalized on patients’ normalized anatomical images and was separated in half. This led to a modified classification scheme with two features per hemisphere. The features were connectivity values which were estimated and extracted as follows: (a) whole‐brain connectivity was estimated using six seed ROIs (R precentral gyrus (*x* = 58, *y* = −6, *z* = 11), R superior temporal gyrus (*x* = 44, *y* = −6 *z* = 11), L precentral gyrus (*x* = −53, *y* = −6, *z* = 8) L superior temporal gyrus (*x* = −44, *y* = −6, *z* = 11), L occipital cortex (*x* = −6, *y* = −83, *z* = 43), R occipital cortex (*x* = 6 *y* = −83, *z* = 43), (b) with the REX toolbox (www.nitrc.org/projects/rex/) these maps as used as Sources and the half brain masks (containing left and right temporal and occipital regions) as ROIs to extract cluster‐level averaged connectivity values for each hemisphere, leading to two features per hemisphere. The classifier was trained on 26 patients in MCS (21 males; mean age = 46 years; 13 traumatic, 13 nontraumatic of which three were anoxic; 20 patients assessed > 41 month postinsult), and 19 patients in VS/UWS (12 males; mean age; one traumatic, 18 nontraumatic of which 11 anoxic; 13 patients assessed 41 month postinsult). The discrimination performance was summarized with the area under the curve (AUC) calculated from the receiver operator characteristic (ROC) curve. For a binary classification system, the ROC pits the detection probability (sensitivity) against the probability of false alarm (1 − sensitivity). These probabilities were empirically estimated by moving the decision cut‐off along the sorted values of a continuous variable and by evaluating its relation to the true label. The AUC was then used to summarize the performance, where a score of 0.5 equals to random guessing, a score of one amounts to perfect classification, and zero to total confusion. The probability of belonging to MCS was estimated by fitting the distribution of the samples with regard to the optimal linear combination of features (*w*).^39^ A sigmoid function was fitted from the distributions of the signed distances separating the train samples and *w*. This sigmoid fit was eventually used to monotonically transform the signed distance separating the test samples and *w* into a meaningful probability. The code for the consciousness level test is openly accessible at https://github.com/fraimondo/hemisphero.

### 2.3 ASL preprocessing and analysis

Due to the poor spatial resolution of the ASL images, registration was performed in an indirect but robust way including the preprocessing of structural T1 data.^40^ Preprocessing steps were achieved using the Voxel Based Morphometry toolbox (VBM8)^41^ as implemented in MATLAB (MathWorks Inc). First, T1 and ASL data are converted from DICOM to NIFTI format. Then, native T1 images are segmented into gray matter, white matter, and cerebrospinal fluid classes. For some patients, when the segmentation failed in the operated hemisphere due to large defects in the white matter, it was necessary to fill the removed area with a “simulated white matter signal” (corresponding to a Gaussian distribution with the same mean and standard deviation intensities than the white matter in the contralateral hemisphere) and segment this new image with VBM8. With the GM and WM segmentation images, a brain mask was built to extract the brain from the native T1 image followed by normalization on a seven‐year‐old‐brain atlas obtained with Template‐O‐Matic Toolbox TOM8 (www.neuro.uni‐jena.de/software/tom) as implemented in SPM8.^42^ ASL images were coregistered on the native GM image to take into account the potential movement of patient during T1 and ASL acquisition including translation and rotation. The coregistered ASL was then normalized using the deformation field obtained during the T1 normalization process. Eventually, the normalized ASL images are smoothed using a 10‐mm isotropic filter. Voxel‐based analyses were performed on smoothed and normalized ASL images within a GM mask in under SPM8, as previously.^40^ At the individual level, voxel‐based analysis was performed using the general linear model, comparing the patient to a control group of 30 healthy pediatric controls according to a methodology previously described.^25^ Results were interpreted with a significance level set at whole‐brain *P* = .05 FWE, and *P* = .001 uncorrected.

## 3 RESULTS

### 3.1 Interhemispheric connectivity

**FIGURE 2:**
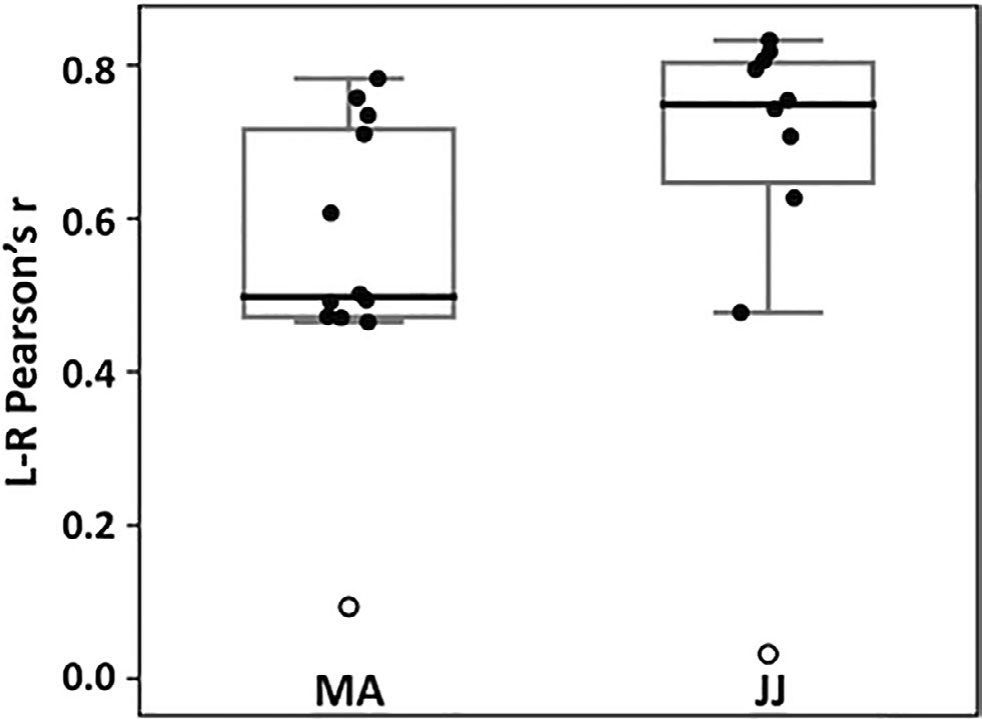
Interhemispheric connectivity was disrupted in patients MA and JJ following complete hemispheric disconnection. Both patients showed correlation values just above zero and appeared as outliers (white circles) among their control subjects (MA controls, n = 11; JJ controls, n = 9). Boxplots represent median (thick line), interquartile range, and minimum and maximum values

Interhemispheric functional connectivity was disrupted in both patients. Pearson's *r* correlations between each hemisphere's GM signal were just above zero for MA (*r* = .09) and JJ (*r* = .03) who both appeared as outliers among their controls (controls MA median: 0.50, min: 0.46, max: 0.78, 1st quartile: 0.48, 3rd quartile: 0.72; controls JJ median: 0.75, min: 0.48, max: 0.83, 1st quartile: 0.71, 3rd quartile: 0.81; Figure 2).

### 3.2 Subcortical level

**FIGURE 3:**
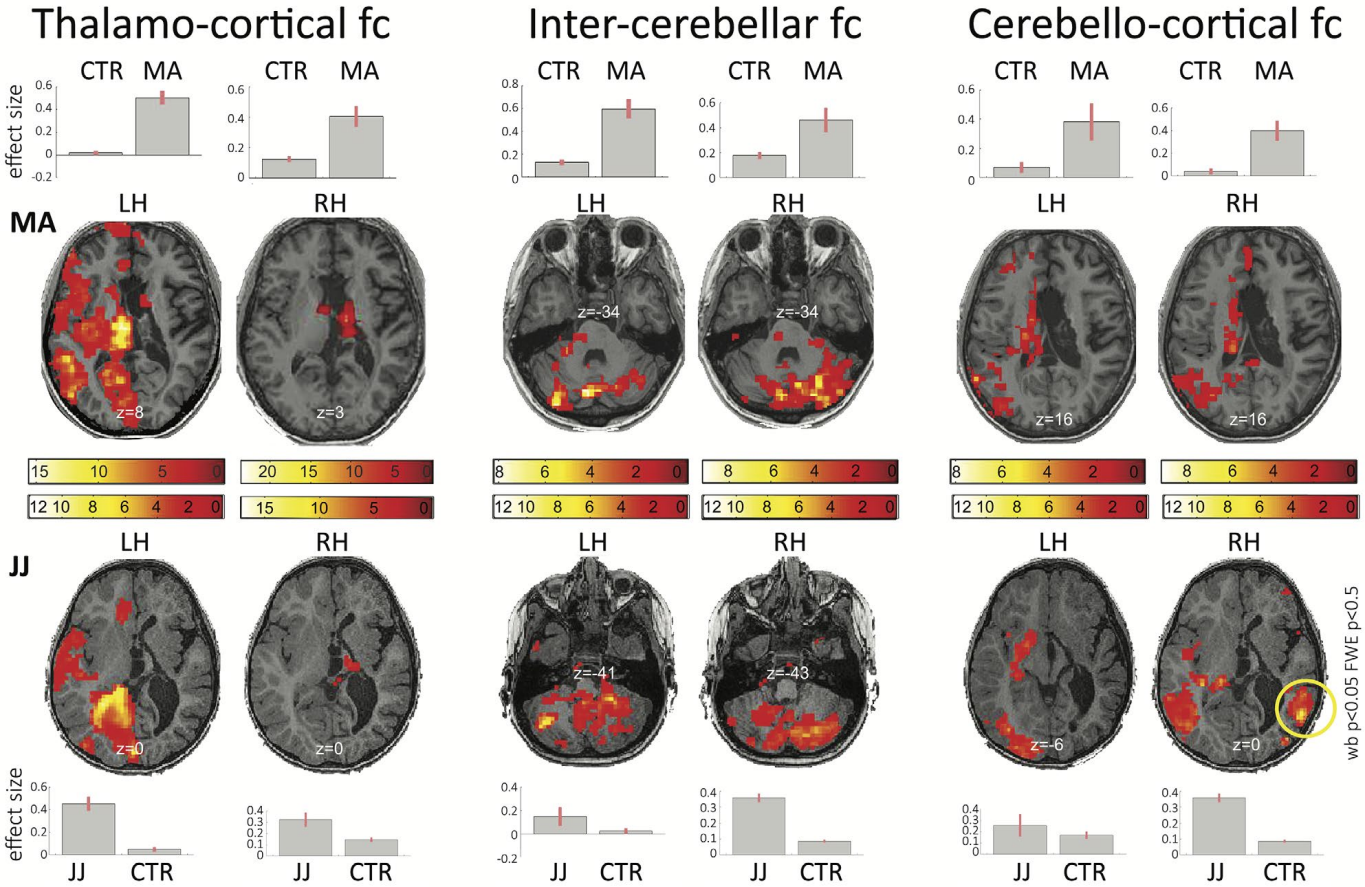
Mediation of subcortical structures in functional reorganization after complete hemispherotomy. Left: Thalamo‐cortical functional connectivity (fc) increased ipsilaterally in the healthy left hemisphere (LH) in both patients, whereas the disconnected right hemisphere (RH) showed no residual thalamo‐cortical connections. Middle: Both patients showed preserved and enhanced connectivity between the two cerebelli. Right: Additionally, the two cerebelli had functional connections with the healthy left cerebral hemisphere but not with the isolated right. The atypical right‐sided ipsilateral cerebello‐cortical connectivity (yellow circle) seen in patient JJ was found to be an artifact mediated by the effect of the vascular system (superior sagittal sinus) and disappeared after regressing out that signal. Statistical maps are thresholded at whole‐brain height threshold *P* < .01 and cluster‐level FWE *P* < .05. Results are rendered on each patient's normalized T1 image. Color bars indicate *t* values. Bars indicate cluster‐level contrast estimates (effect size) with 90% confidence intervals. Numbers in white refer to MNI slice coordinates (axial view). CTR, healthy control subjects

Subcortically, patients showed increased left ipsilateral thalamo‐cortical connectivity, whereas the disconnected right hemisphere showed no residual thalamo‐cortical functional connections. Both patients had increased connectivity between the two cerebelli, and between the healthy left cerebral hemisphere and both cerebellar hemispheres (Figure 3). Patient JJ showed further right‐sided ipsilateral cerebello‐cortical connectivity, which was atypical given the surgical disconnection (Figure 3, yellow circle). As the vascularization system is a major physiological system shared by the two hemispheres, it was hypothesized as the main source of this atypical cortico‐subcortical correlation. Indeed, considering the SSS as a seed region, statistical association was predicted between the SSS signal primarily the disconnected right hemisphere (Figure S1).

### 3.3 Lateralized functional organization

**FIGURE 4:**
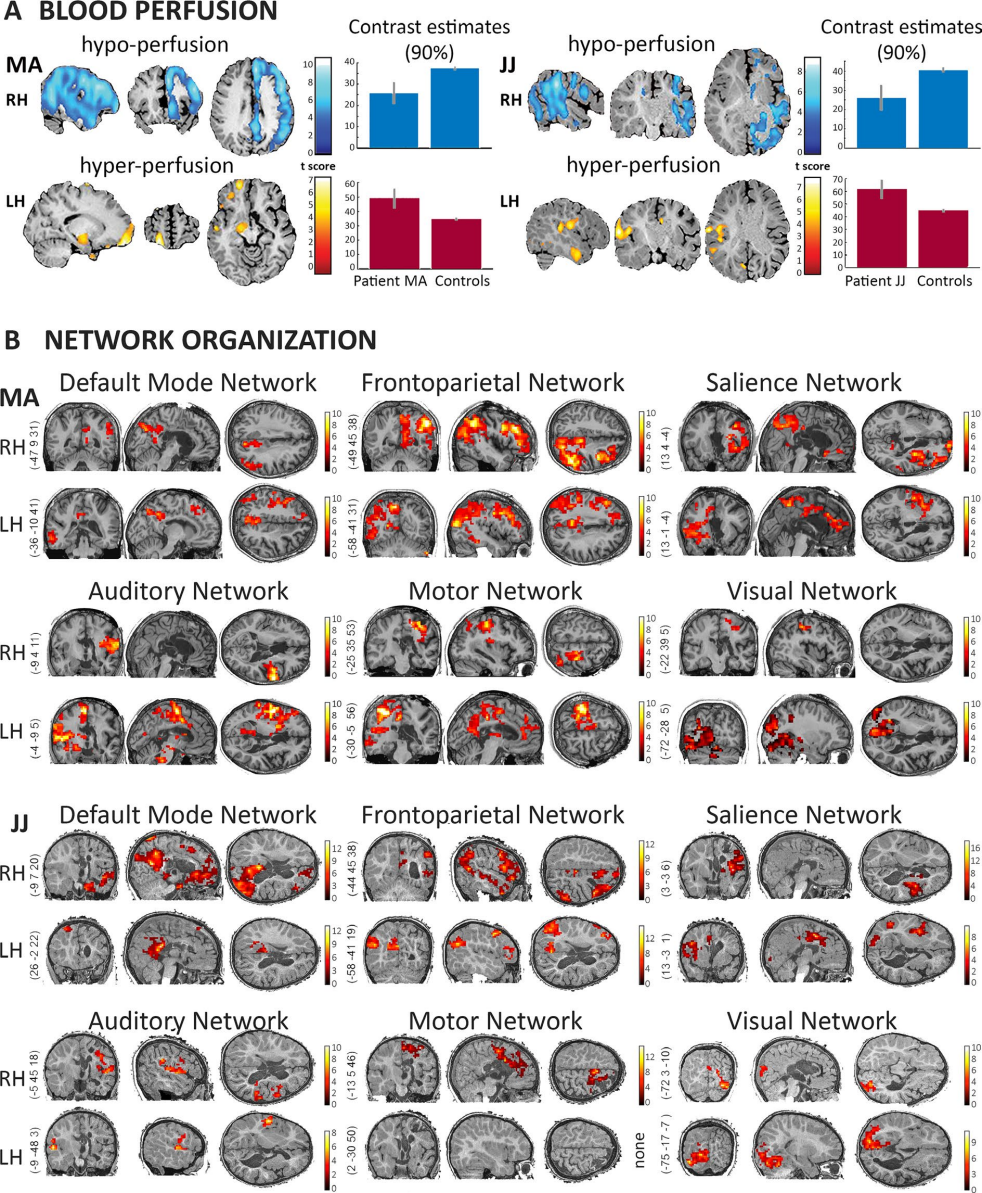
Whole‐brain functional organization was laterized after complete hemispherotomy. A, In terms of blood perfusion, cerebral blood flow was lower within the disconnected right hemisphere (blue regions, FWE *P* < .05) compared to healthy controls both for patient MA and JJ. At the same time, focal hyperperfusion was observed in the remaining left hemisphere in both patients (blue regions, FWE *P* < .05) compared to healthy controls. Bars represent averaged contrast estimates across the identified cluster with 90% confidence interval (whiskers). The statistical maps are rendered on the patient's normalized T1 image. B, In terms of functional connectivity, both patients showed network organization in six representative systems. The connectivity appeared lateralized within the right (RH) and left hemisphere (LH) where the seeds were located and did not show contralateral connectivity transfer. Of note is the preserved yet restricted network‐level connectivity in the isolated right hemisphere even after the regression of the vascularization effect of the superior sagittal sinus. Statistical maps are thresholded at whole‐brain height threshold *P* < .01 and cluster‐level FWE *P* < .05. Results are rendered on each patient's normalized T1 image. Color bars indicate *t* values. Side numbers refer to MNI slice coordinates

The disconnected right hemisphere showed significant (*P* < .05, FWE‐corrected) diffuse decreases in cerebral blood flow (CBF) values in both patients compared to healthy individuals (MA, mean = 16.7 mL/100 mg/mn ± 15.4SD; JJ, mean = 34.1 ± 17.3SD; controls mean = 45 mL/100 mg/mn ± 2.7SD; FWE‐corrected *P* = .05; Figure 4A). Crossed‐cerebellar hypoperfusion was also noted in both patients. After regressing out the effect of the SSS, fMRI functional connectivity in the disconnected right hemispheres showed persisting lateralized network‐level organization in large‐scale (default mode, frontoparietal, salience) and sensory systems (auditory, motor, visual). Seed ROIs placed on the right hemisphere showed no contralateral connectivity in either patient (whole‐brain *P *< .01, cluster‐level FWE *P* < .05; Figure 4B), in contrast to the bilateral connectivity typically observed in healthy controls (Figure S3 and Figure S4).

The healthy left hemisphere showed localized significant (*P* < .05, FWE‐corrected) increases in CBF values in both patients compared to healthy individuals (MA, mean = 66.2 mL/100 mg/mn ± 10.5SD; JJ, mean = 69.05 mL/100 mg/mn ± 11.6SD, controls mean = 47 mL/100 mg/mn ± 1.8SD). Hyperperfusion was located in the motor operculum, amygdala, temporal and frontal pole in MA and in the temporal pole and sensorimotor operculum in JJ (Figure 4A). After regressing out the effect of SSS, fMRI functional connectivity showed lateralized network‐level organization in large‐scale (default mode, frontoparietal, salience) and sensory systems (auditory, motor, visual). Seed ROIs placed on the left hemisphere showed no contralateral connectivity in either patient (whole‐brain *P* < .01, cluster‐level FWE *P* < .05; Figure 4B) in contrast to the bilateral connectivity typically observed healthy controls (Figure S3 and Figure S4). The unthresholded network maps for the two patients can be accessed at https://identifiers.org/neurovault.collection:8380

### 3.4 Classification of consciousness level

**FIGURE 5:**
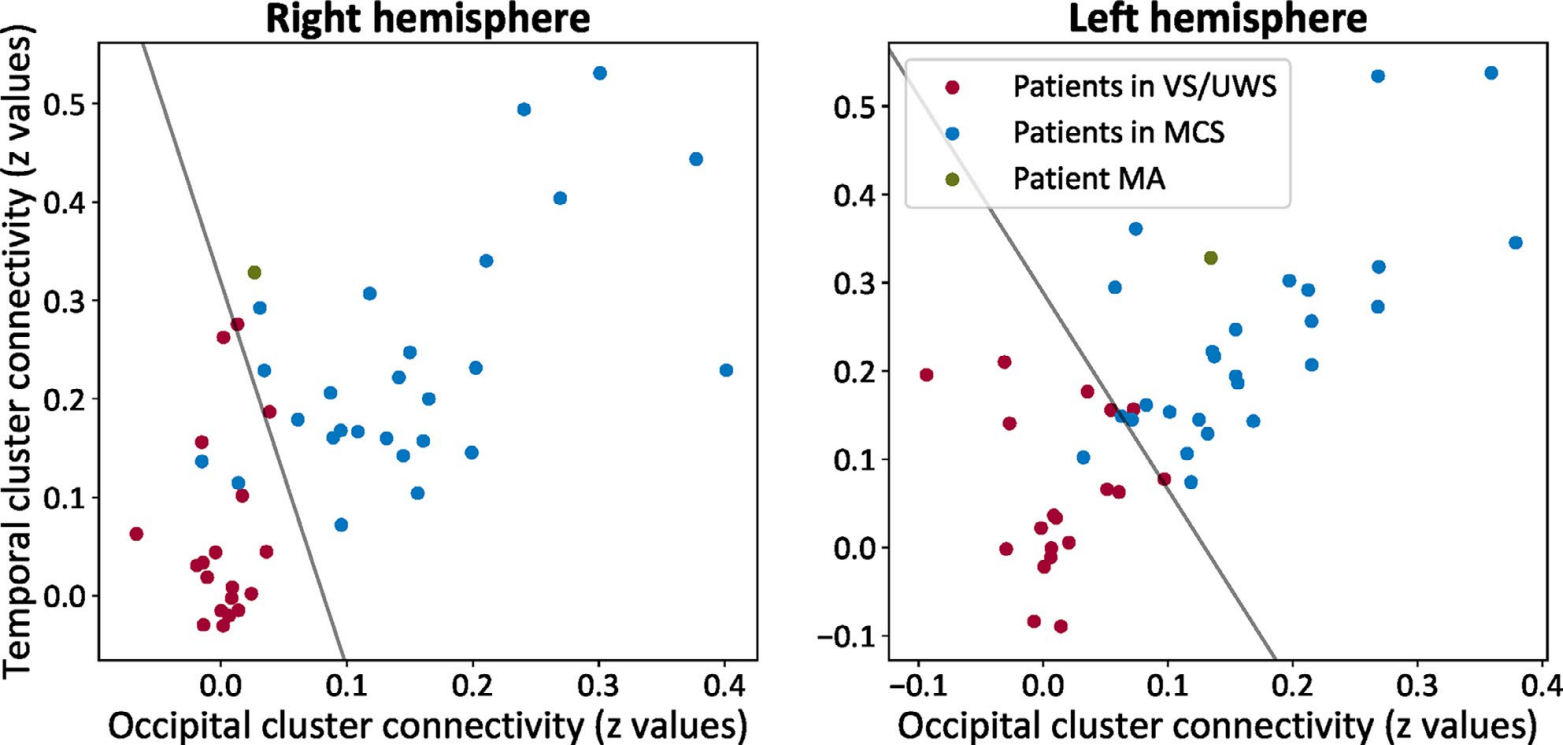
The contribution of each hemisphere to the state of consciousness. For patient MA (green), the isolated right hemisphere was closer to the class of patients in vegetative state/unresponsive wakefulness syndrome (VS/UWS, in red; showing reflexive behaviors) and had low chances to be classified among patients in minimally conscious state (MCS, in blue, showing complex behaviors to external stimulations but who remain unable to communicate). At the same time, the preserved left hemisphere was classified toward the class of MCS with higher probability. The line represents the decision boundary between the two classes as estimated by the linear support vector classifier

The classification included patient MA as he was the most comparable to the included subjects in the training set. For the preserved left hemisphere, the AUC for the occipital region was 0.97 and for the right temporal region was 0.88—the probability of belonging to the class of MCS was 0.96. For the isolated right hemisphere, the AUC for the occipital region was 0.94 and for the right temporal region was 0.88—the probability of belonging to the class of MCS was 0.65 (Figure 5).

## 4 DISCUSSION

We quantified whole‐brain functional organization in two pediatric patients treated with hemispherotomy for intractable epilepsy, aiming to address scientific and clinical concerns raised by this rare surgical intervention. Broadly, we found that both patients exhibited preserved blood perfusion and network organization, of a clearly lateralized pattern. Also, their disconnected hemispheres were characterized by a lower similarly to MCS by comparison to the healthy right hemisphere.

### 4.1 Breakdown of interhemispheric connectivity

We identified that interhemispheric functional connectivity was disrupted in both patients, who nevertheless retained their overall behavioral performance. Interhemispheric communication is a critical feature of the brain's organization for promoting a balanced transfer of information.^14^ However, the disconnection of the two hemispheres as a result of a surgical intervention does not seem to affect the overall behavioral and cognitive functioning of the treated children. This is particularly evident for hemispherectomies, during which the entire epileptic tissue is resected, leading to language reorganization^43^ and to retained functional connectivity in the remaining hemisphere.^44^ Therefore, the breakdown of interhemispheric functional connectivity transfer after hemispherotomies is consistent with the surgical procedure which aimed at disrupting the interhemispheric communication in the first place.

### 4.2 Cortico‐subcortical organization

We also found that blood perfusion and connectivity increased in the healthy left cortex and subcortical structures. The increased left thalamo‐cortical connectivity was expected as the thalamus is a major node in brain networks.^45^ This postoperative connectivity pattern was similar to a previous report on a hemispherectomy where decreased connectivity ﻿in the thalamo‐default mode network within the remaining hemisphere was detected, providing direct evidence that functional interactions depend on structural connections.^2^ As such, we postulate that high‐order thalamic nuclei may be key drivers for functional adaptation in the remaining hemisphere.

This hypothesis is further supported by electrophysiological recordings in nonhuman primates and rodents where thalamic high‐order relay can control connectivity and modular organization.^46^, ^47^ The identified enhanced crossed cerebello‐thalamo‐cortical connectivity is also in agreement with previous imaging studies after cortically extended lesions.^48^ These findings are related to anatomical modifications that were noted both in animal models and after hemispherectomy, with expansion of the afferent and efferent fibers of the cortico‐ponto‐cerebello‐rubro‐thalamic system.^49^, ^50^ As the cerebellum is highly involved in learning^51^ and as it modulates cerebral excitability via its thalamic inhibition,^52^, ^53^ we hypothesize that after hemispherotomy increased connectivity between cerebellar hemispheres shapes cortical organization. Our hypothesis was based on previous findings that after left hemispherotomy language organization takes place in the right hemisphere.^11^ Recently, it also was shown that a split‐brain patient exhibited increased fractional anisotropy of the dorsal and ventral pontine decussations of the cortico‐cerebellar interhemispheric pathways, suggesting that cerebellar anatomical substrates may account for the spared interhemispheric coordination and intact cognitive abilities.^22^ Moreover, the cerebellum's role in motor recovery may further involve bilateral spinal efferences through the Ruber nuclei to compensate the loss of the cortico‐spinal tract as already demonstrated in animal models and after stroke in humans.^54^, ^55^

### 4.3 Functional connectivity and vascularization

We identified that the atypical right‐sided ipsilateral cerebello‐cortical connectivity in patient JJ was mediated by the effect of the vascular system. Also, this ipsilateral right connectivity disappeared after regression of the SSS signal, which suggests that nonneuronal signals echo in the isolated tissue and might contribute to network organization which to date is widely considered of neuronal origin.^31^, ^56^, ^57^ Indeed, the here identified rCBF reductions along with preserved network organization in the disconnected right hemisphere were beyond our hypothesis. The question as to why intrinsic network functional connectivity is preserved in isolated tissue which does not contribute to behavioral output, remains to be answered. A working hypothesis is that brain activity is driven by the underlying anatomy as we have recently found for noncommunicating states.^32^ At the same time, it may be that preserved network‐level connectivity is critical for the development of synaptic connections and maintenance of synaptic homeostasis at large^58^ which is reduced, yet preserved, in covert or unconscious conditions.^32^, ^57^

### 4.4 Intra‐hemispheric participation to the level of consciousness

The disconnected right hemisphere might therefore be a model of unilateral disorder of consciousness.^59^ For instance, global rCBF decreases were noted before in patients in MCS.^60^ Also, reductions in connectivity strength within large‐scale and sensory networks were identified in these patient groups.^32^, ^61^, ^62^ These observations raise queries as to the role of such intrinsic organization in isolated brain tissue. As suggested by the here tested classification test, the similarity of patient's MA left hemisphere was closer to that of the MCS class, whereas his right hemisphere was less comparable to this group. This finding is in line with our hypothesis that the isolated hemisphere might contribute less to consciousness, and that thalamo‐cortical processing plays a necessary role for conscious processing. Also, note that MCS has been recently reinterpreted as a cortically mediated state,^63^ indicative more of a class of behaviors revealing an active contribution of cortical networks, rather than a univocal conscious state. Under such hypothesis, MCS does not relate to consciousness but to a necessary but insufficient condition for conscious processing. The classification of the level of consciousness then should be interpreted mostly as indicative information rather than an absolute model for consciousness function in the isolated brain tissue.

### 4.5 Neurological mechanisms

In the healthy left hemisphere, we identified rCBF increases in the somatosensory cortex, and mesial/lateral temporal region compatible with elevated neuronal activity. These results are in line with a previous TMS study after hemispherotomy showing enhanced motor cortex excitability after surgery.^5^ Following the lesion paradigm of stroke, upregulation of contralateral homotopical areas has already been reported in the left middle cerebral artery territory, where aphasic patients showed this upregulation in the right Broca‐homologue region during language tasks in the subacute phase.^64^ Such a phenomenon could rely on the disinhibition of the healthy hemisphere. After hemispherotomy, this disinhibition could be indeed related to the callosotomy despite that most of callosal fibers transmit excitatory glutamatergic inputs. In rodents after contralateral sensory stimulation of the somatosensory cortex, the firing of layer 5 pyramidal neurons is inhibited when paired with ipsilateral stimulation, suggestive of interhemispheric inhibition.^65^ The localization of the focal rCBF increases is further reminiscent of activation tasks in fMRI and neurophysiological studies after hemispherotomy, pointing to residual function originated from the remaining healthy hemisphere in expected networks. As such, magnetoencephalographic somatosensory evoked potentials elicited ipsilateral responses in the primary somatosensory cortex in three patients with residual sensory function after hemispherectomy.^66^ Combined neurophysiological and fMRI data showed ipsilateral activation of the sensorimotor region during passive movement of the hand in a location similar to the movements of the other hand, yet with a greater spatial extent^8^ ^.^ Taken together, the preserved left hemispheric increased perfusion might reflect a mechanism of disinhibited neural activity after the surgery.

In the disconnected right hemisphere, we identified reductions in rCBF both ipsilaterally and with the contralateral cerebellum. Reduced hemispheric metabolic demands have been previously described as secondary to thalamic stroke and after thalamotomy for tremor.^67^, ^68^ After hemispherotomy, section of the perithalamic white matter tracts suppresses seizure expression due to the interruption of the cortical projection bundles to the brainstem and spinal cord and, in the meantime, interrupts the ascending reticular activating system, an essential polysynaptic pathway for arousal through increases of cortical excitability.^69^ Crossed‐cerebellar and ipsilateral diaschisis were also recently reported with reduced perfusion of rCBF after thalamic or putaminal hemorrhage.^70^ Such significant decreases in neuronal metabolism may be due to reduced neuronal activity secondary to the loss of afferent inputs as in rodents cerebellar interneurons and Purkinje cells decrease their firing rate after inducing focal cerebral ischemia.^71^

### 4.6 Study limitations

A direct limitation of our study is the small patient number. Inherently with all case studies, our results are therefore prone to biases when generalized to the population. Also, the absence of a follow‐up protocol does not permit to draw definite conclusions about the cerebral organization after hemispherotomy at long term. Furthermore, in order to compensate for the lack of available controls during our protocol, we resorted to the open National Database for Autism Research (NDAR) for recruiting subjects matched for age with our two patients. By doing so, we compromised having insufficient information about the intellectual functioning and handedness; also, the control subjects were evaluated in variant MRI settings (different scanners and scanning parameters). We nevertheless justify our choice by considering that the control data meant to work as a visual reference for illustrating the connectivity effects of the performed analyses rather than used for direct statistical comparisons with our patients. Although this aim balances the aforementioned challenges, we naturally recognize that more controlled data collection is needed for referenced groups. Finally, the classification of the level of consciousness should be interpreted mostly as indicative information rather than an absolute model for consciousness function. This is because the used classifier was formed on a qualitatively different population to separate the state of consciousness, namely adult patients suffering severe brain damage. Considering, though, the sparsity in the number of patients having received complete hemispherotomy, we think that even such coarse testing sheds light on the ongoing debates about the role of the isolated hemisphere in awarenesss^13^ and paves the way for more thorough examination of the preserved capacities of isolated brain tissue by more perturbational means that can inform the capacity for conscious processing.^13^, ^72^

In conclusion, after complete hemispherotomy whole‐brain functional organization appears lateralized, allowing us to postulate that in the healthy hemisphere, cortical disinhibition and enhanced connectivity, driven by subcortical structures through preexisting networks, mediate neurological recovery. Our results point to the importance of anatomical connectivity driving the presence of network‐level organization, highlight the prominence of the vascular system in functional connectivity after hemispherotomy and pave the way for more targeted assessment of cognitive/conscious state in the isolated hemisphere.

We would like to acknowledge that the study has been previously reported in biorxiv with the following reference https://www.biorxiv.org/content/10.1101/707539v1.

## CONFLICTS OF INTEREST

The authors report no relevant conflict of interest. The authors confirm that they have read the journal's position on issues involved in ethical publication and affirm that this report is consistent with those guidelines.

## Supporting information

Supplementary Material
