# Supplementary material for: Complete hemispherotomy leads to lateralized functional organization and lower level of consciousness in the isolated hemisphere
Source: Epilepsia Open. 2020 Sep 24;5(4):537–49. doi: 10.1002/epi4.12433 (PMC7733653; doi:10.1002/epi4.12433)
Supplement: Supplementary file 1 — Supplementary Material [file EPI4-5-537-s001.pdf]

Supporting Information

Complete hemispherotomy for intractable epilepsy leads to bilaterally preserved functional organization

Thomas Blauwblomme[1,2,3]*†, Athena Demertzi[4,5,6]†, Jean-Marc Tacchela[3], Ludovic Fillon[3], Marie Bourgeois[1], Emma Losito[1], Monika Eisermann[1], Daniele Marinazzo[7],Federico Raimondo[6,8], [9]Sarael Alcauter, Frederik Van De Steen[4], Nigel Colenbier[4], Steven Laureys[8], Volodia Dangouloff-Ros[1,2,3], Lionel Naccache[5,6], Nathalie Boddaert[1,2,3], Rima Nabbout[1,2,3]

†These authors contributed equally to this work

[1]Assistance Publique Hôpitaux de Paris, Hôpital Necker-Enfants Malades, Paris, France ; [2]Université de Paris, Paris, France ; [3]Institut Imagine, INSERM U1163, Paris, France ; [4]GIGA-Consciousness, Physiology of Cognition Research Lab, GIGA Institute, University of Liège, Belgium ; [5]INSERM, U1127, Paris, France; [6]Institut du Cerveau et de la Moelle Epinière, Hôpital Pitié-Salpêtrière, Paris, France ; [7]Department of Data Analysis, Faculty of Psychological and Educational Sciences, University of Ghent, Belgium; [8]GIGA-Consciousness, Coma Science Group, GIGA Institute, University of Liège, Belgium; [9]Instituto de Neurobiología, Universidad Nacional Autónoma de México, Querétaro, México

***Corresponding author:**

Thomas Blauwblomme

Hôpital Necker-Enfants Malades, 149 rue de Sèvres 75015 Paris, France

Phone number: +33 1 71396593

Email: thomas.blauwblomme@aphp.fr

Figure S1. Explaining the cerebello-cortical functional connectivity of the pathological hemisphere. The vascularization system was hypothesized as the main source of inter-hemispheric transfer of cortico-subcortical functional connectivity as it constitutes a major physiological system shared by the two hemispheres. Considering the superior sagittal sinus (SSS) as a seed region, statistical associations were predicted in bilateral hemispheres. Statistical maps are thresholded at whole-brain height p<0.01, FWE cluster level p<0.05 and rendered on each patient's normalized MRI.

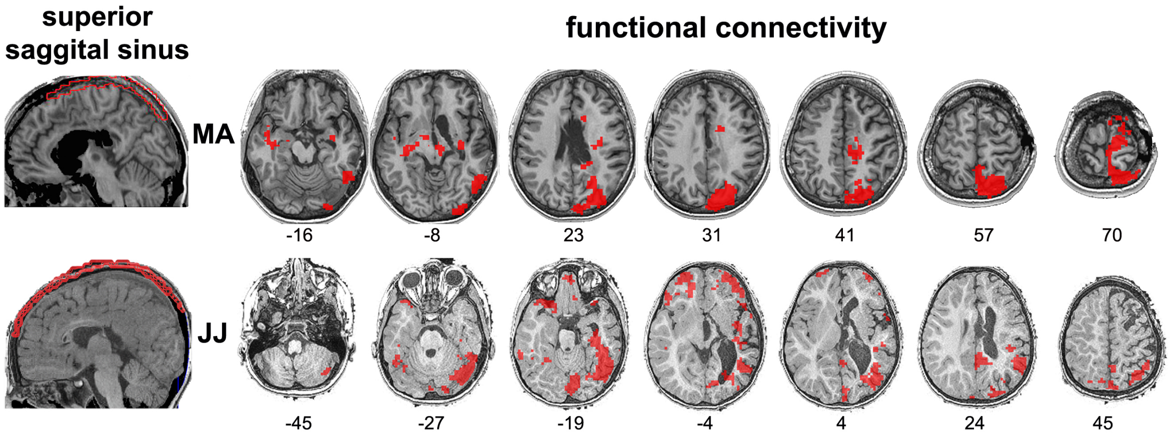

Figure S2. To verify this effect on patient JJ, two validation tests were performed. First, we noted that original cerebellar seed (blue) was positioned over the functional connectivity (fc) which was predicted by the SSS timeseries (red region). When a new cerebellar seed was used (green), not overlapping with the SSS effect, no ipsilateral right connectivity was observed. Second, when two other seeds (yellow, over the inferior frontal gyrus-IFG and the inferior temporal gyrus-ITG) were placed on regions which were functionally connected with the SSS timeseries, both ipsi- and contralateral connectivity was predicted, verifying the confounding effect of the vascularization of the SSS on the isolated right hemisphere. Statistical maps are thresholded at whole-brain height $p<0.01$, FWE cluster level $p<0.05$ and rendered on each patient's normalized MRI.

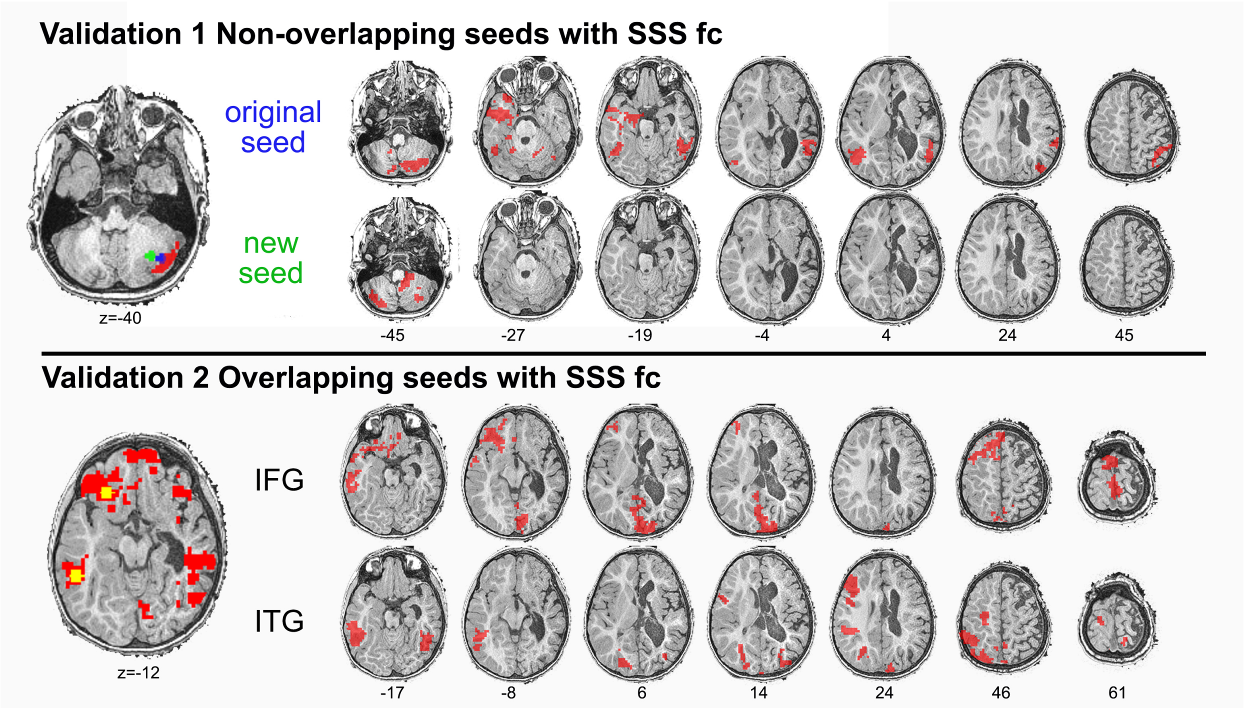

Figure S3. Network-level organization of intrinsic functional connectivity in the right and left hemisphere as estimated in patient MA and his healthy control subjects (CTR, n=11). Of note is the bilateral connectivity in the control group for right- and left-sided regions of interest. This interhemispheric connectivity effect was not observed in patient MA, who showed lateralized connectivity restricted to each hemisphere for the six studied networks. Statistical maps are thresholded at whole-brain height threshold p<0.01 and at FWE p<0.05 (cluster-level correction) and rendered on a stereotaxic template (MRIcron, ch2 template) with coronal, sagittal and axial views. Colourbars indicate t values. Bottom numbers refer to MNI slice coordinates.

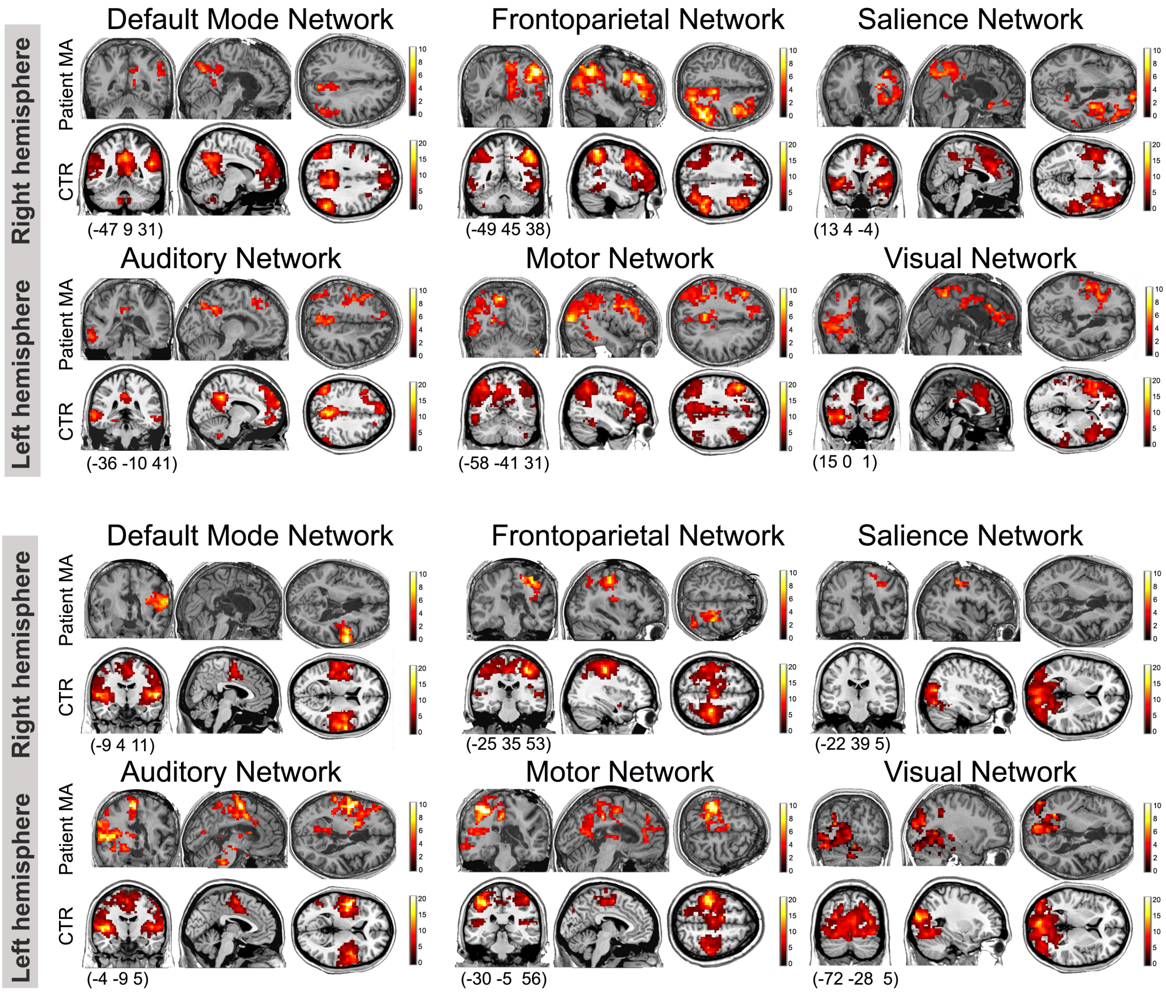

Figure S4. Network-level organization of intrinsic functional connectivity in the right and left hemisphere as estimated in patient JJ and his healthy control subjects (CTR, n=9). Of note is the bilateral connectivity in the control group for right- and left-sided regions of interest. This interhemispheric connectivity effect was not observed in patient JJ, who showed lateralized connectivity restricted to each hemisphere for the six studied networks. Statistical maps are thresholded at whole-brain height threshold p<0.01 and at FWE p<0.05 (cluster-level correction) and rendered on a normalized 2 year old infant template with coronal, sagittal and axial views. Colourbars indicate t values. Bottom numbers refer to MNI slice coordinates.

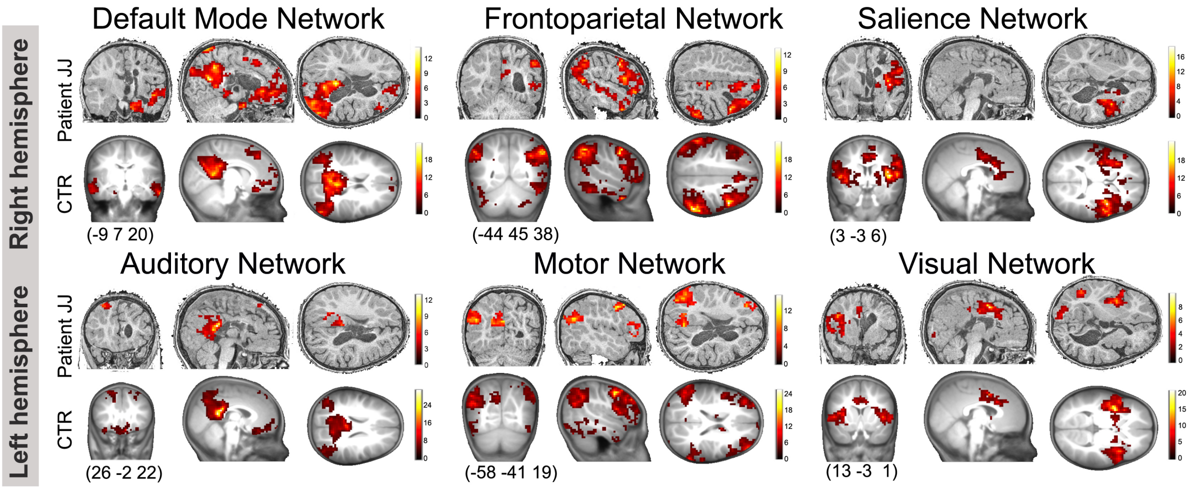

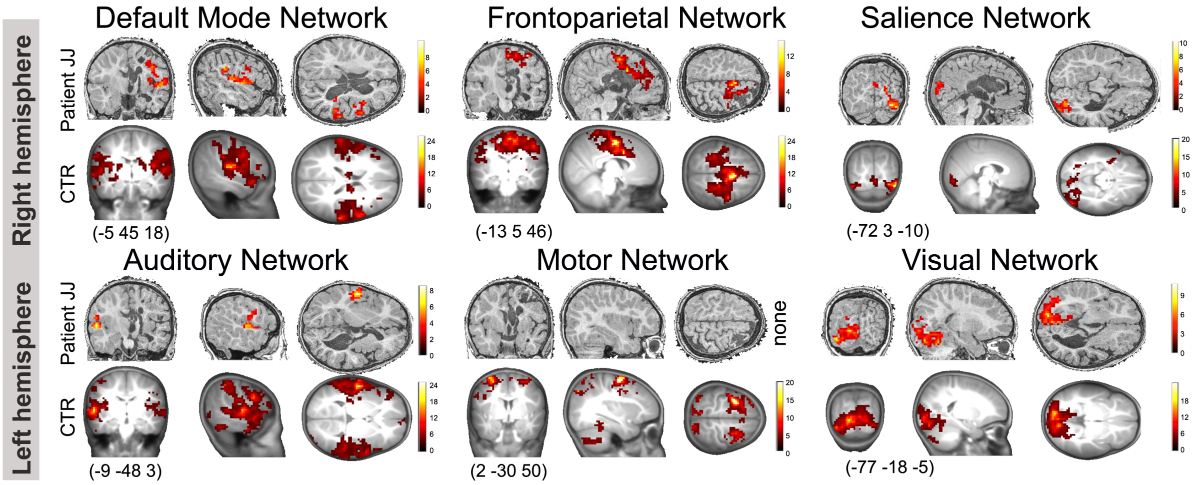

Figure S5. Registration output- regions of interest were manually designed following the patient's anatomical constraint (patient MA; DMN: default mode network, CER: cerebellum)

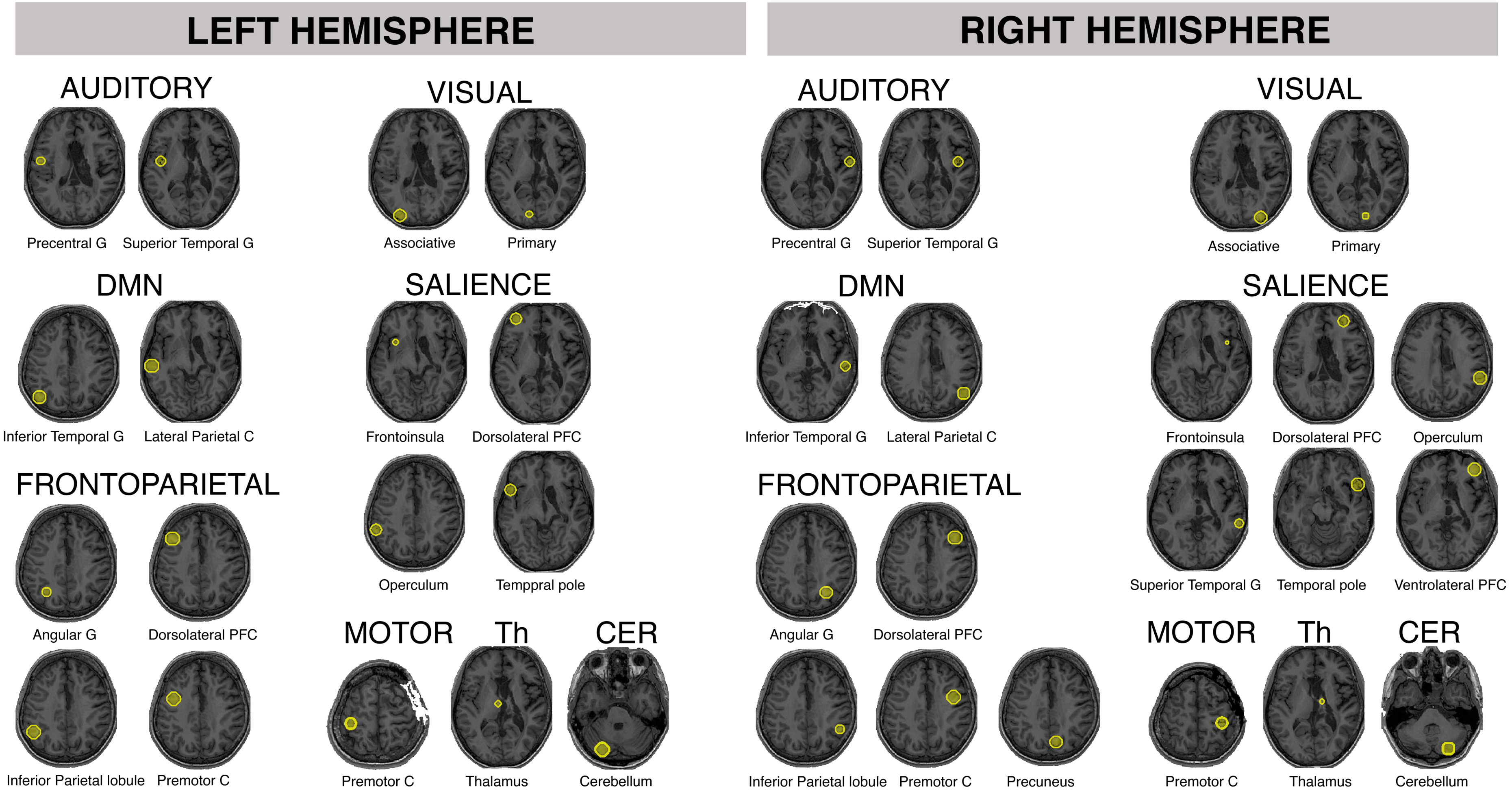

Figure S6. Registration output- regions of interest were manually designed following the patient's anatomical constraint (patient JJ; DMN: default mode network, CER: cerebellum).

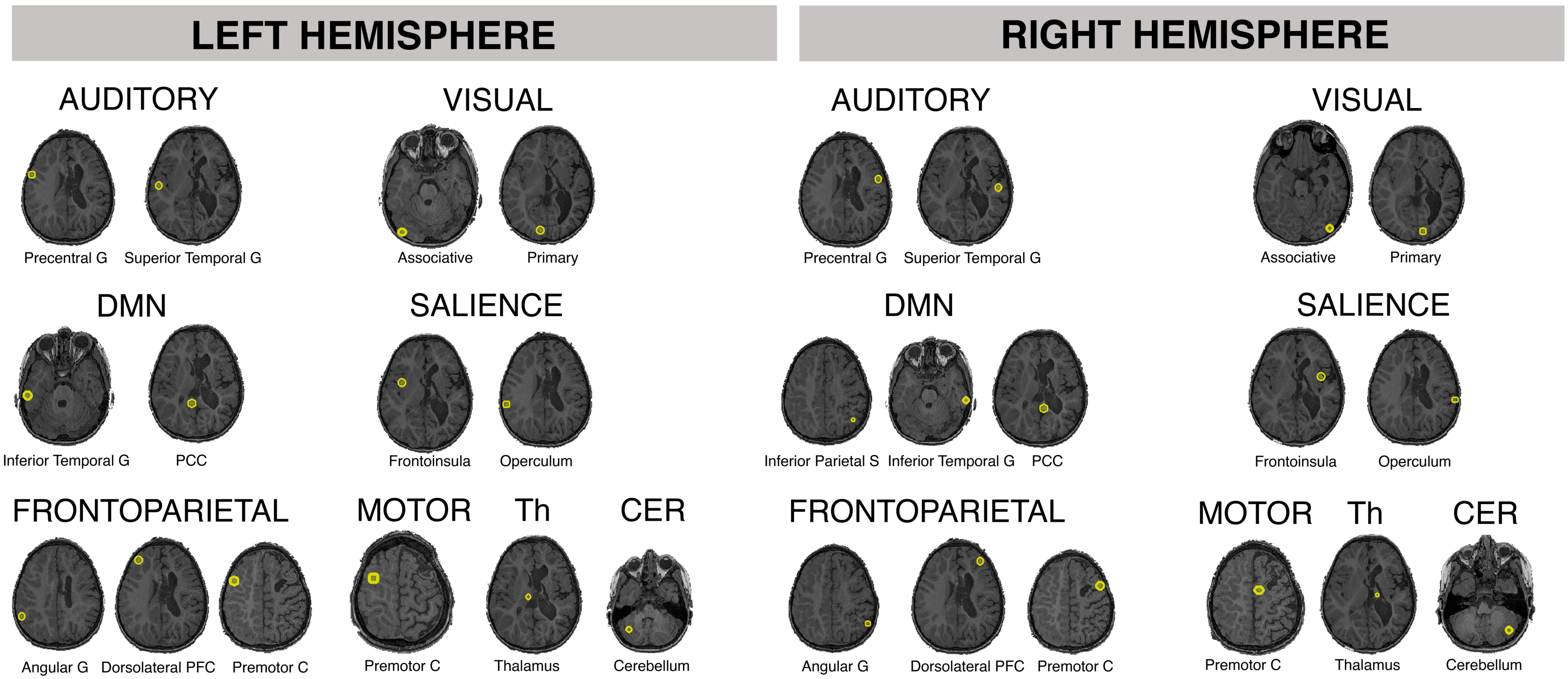

Figure S7. Denoising results. In our analysis the denoising procedure encompassed: 1) motion artifact detection using the artifact detection toolbox (ART); 2) regressing out the realignment parameters, their derivatives and the ART-detected motion outliers, 4) regressing our the signal from the superior sagittal gyrus, and 4) an anatomical component-based noise correction method (aCompCor) which models the influence of noise as a voxel-specific linear combination of multiple empirically estimated noise sources, such as white matter, gray matter and cerebrospinal fluid. Such a denoising procedure is known to lead to a distribution of the seed-to-voxel correlation values around zero (Chai et al., 2012; NeuroImage 59 (2): 1420–28). The results of the employed denoising steps are summarized below.

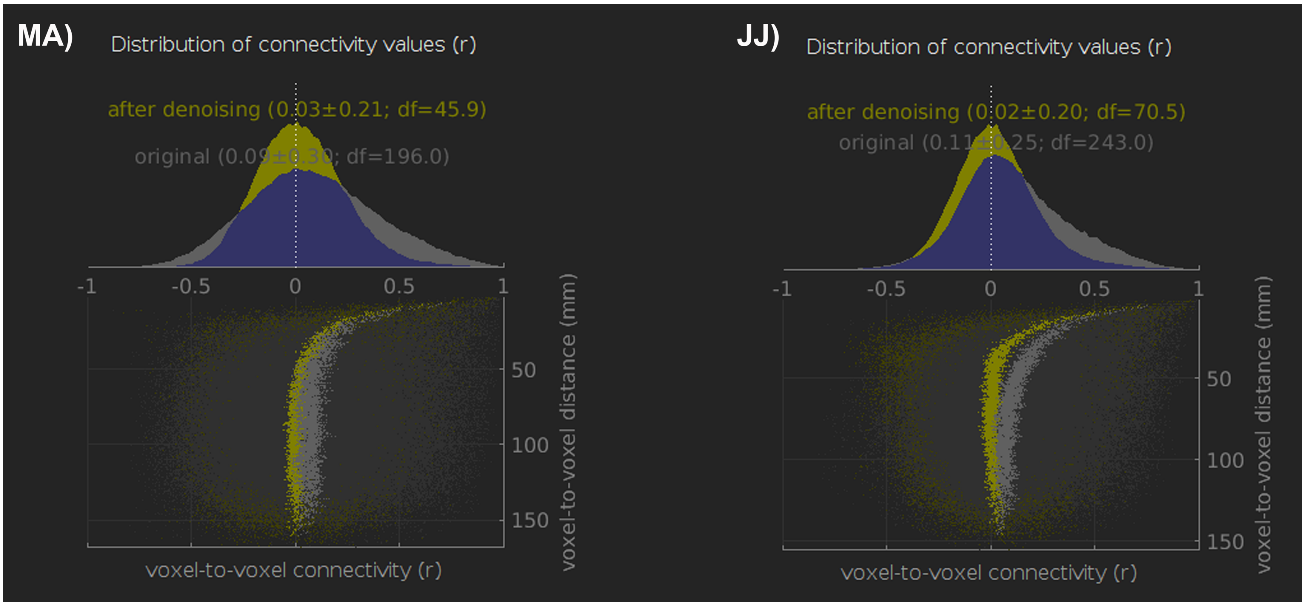

Table S1. Regions of interest for patient MA. Regions of interest were spheres (5mm-radius) designed based on each patient's anatomical constraints referring to the most pertinent intrinsic connectivity networks.

**Position**	**Network**	**Name**	**Coordinates**
R	Auditory	Precentral Gyrus	58 -6 11
R	Auditory	Superior Temporal Gyrus	44 -6 11
R	DMN	Inferior Temporal Gyrus	58 -24 -9
R	DMN	Lateral Parietal Cortex	49 -63 33
R	Frontoparietal	Angular Gyrus	30 -61 39
R	Frontoparietal	Dorsolateral Prefrontal Cortex	43 22 34
R	Frontoparietal	Inferior Parietal Lobe	51 -47 42
R	Frontoparietal	Premotor Cortex	41 3 36
R	Frontoparietal	Precuneus	10 -69 39
R	Motor	Premotor Cortex	38 -26 48
R	Salience	Frontoinsula	39 11 -5
R	Salience	Dorsolateral Prefrontal Cortex	30 48 22
R	Salience	Operculum	58 -40 30
R	Salience	Superior Temporal Gyrus	64 -38 6
R	Salience	Temporal Pole	52 20 -18
R	Salience	Ventolateral Prefrontal Cortex	42 46 0
R	Visual Associative	Visual Associative	30 -89 20
R	Visual Primary	Visual Primary	11 -87 4
L	Auditory	Precentral Gyrus	-53 -6 8
L	Auditory	Superior Temporal Gyrus	-44 -6 11
L	DMN	Inferior Temporal Gyrus	-61 -24 -9
L	DMN	Lateral Parietal Cortex	-46 -66 30
L	Frontoparietal	Angular Gyrus	-31 -59 42
L	Frontoparietal	Dorsolateral Prefrontal Cortex	-43 22 34
L	Frontoparietal	Inferior Parietal Lobe	-51 -51 36
L	Frontoparietal	Premotor Cortex	-41 3 36
L	Motor	Premotor Cortex	-39 -26 51
L	Salience	Frontoinsula	-33 13 -6
L	Salience	Dorsolateral Prefrontal Cortex	-38 52 10

L	Salience	Operculum	-60 -40 40
L	Salience	Temporal Pole	-52 16 -14
L	Visual Associative	Visual Associative	-30 -89 20
L	Visual Primary	Visual Primary	-10 -87 2
L	Cerebellum	Cerebellum	-25 -81 -33
L	Thalamus	Thalamus	-6 -12 4
R	Cerebellum	Cerebellum	25 -81 -33
R	Thalamus	Thalamus	12 -9 2

Table S2. Network-level organization of regions of interest (patient JJ). Regions of interest were spheres (5mm-radius) designed based on each patient's anatomical constraints referring to the most pertinent intrinsic connectivity networks.

Position	Network	Name	Coordinates
R	Auditory	Precentral Gyrus	49 4 24
R	Auditory	Superior Temporal Gyrus	49 -14 6
R	DMN	Inferior Parietal Sulcus	41 -57 38
R	DMN	Inferior Temporal Gyrus	56 -24 -21
R	DMN	Posterior Cingulate Cortex	6 -41 10
R	Frontoparietal	Angular Gyrus	52 -52 38
R	Frontoparietal	Dorsolateral Prefrontal Cortex	33 40 21
R	Frontoparietal	Premotor Cortex	44 8 40
R	Motor	Supplementary Motor Area	6 -7 45
R	Salience	Frontoinsula	36 4 4
R	Salience	Operculum	61 -30 25
R	Visual	Associative Visual Cortex	37 -73 -21
R	Visual	Primary Visual Cortex	9 -79 -1
L	Auditory	Precentral Gyrus	-53 7 23
L	Auditory	Superior Temporal Gyrus	-51 -12 2
L	DMN	Inferior Temporal Gyrus	-52 -22 -27
L	DMN	Posterior Cingulate Cortex	-6 -41 10
L	Frontoparietal	Angular Gyrus	-53 -46 29
L	Frontoparietal	Dorsolateral Prefrontal Cortex	-34 40 24
L	Frontoparietal	Premotor Cortex	-42 7 40
L	Motor	Premotor Cortex	-29 -3 52
L	Salience	Frontoinsula	-34 4 3
L	Salience	Operculum	-57 -33 23
L	Visual	Associative Visual Cortex	-38 -73 -25
L	Visual	Primary Visual Cortex	-10 -78 -4
R	Cerebellum	Cerebellum	33 -55 -38
L	Cerebellum	Cerebellum	-33 -51 -37
R	Cerebellum	Cerebellum not overlapping	25 -53 -37
L	Thalamus	Thalamus	-8 -15 8
R	Thalamus	Thalamus	13 -13 7
